# Characterization of a human thyroid microtissue model for testing thyroid disrupting chemicals

**DOI:** 10.3389/ftox.2024.1408808

**Published:** 2024-07-24

**Authors:** E. Rogers, E. K. Breathwaite, T. Nguyen-Jones, S. M. Anderson, J. J. Odanga, D. T. Parks, K. K. Wolf, T. Stone, P. Balbuena, J. Chen, S. C. Presnell, J. R. Weaver, E. L. LeCluyse

**Affiliations:** ^1^ Research and Development, LifeSciences Division, LifeNet Health, Va Beach, VA, United States; ^2^ Research and Development, LifeSciences Division, LifeNet Health, Research Triangle Park, NC, United States

**Keywords:** thyroid, thyrocyte, thyroxine, thyroglobulin, microtissues, TDC, endocrine disruption, hypothyroidism

## Abstract

Perturbation of thyroid hormone (T_4_) synthesis is known to cause numerous developmental, metabolic, and cognitive disorders in humans. Due to species differences in sensitivity to chemical exposures, there is a need for human-based *in vitro* approaches that recapitulate thyroid cellular architecture and T_4_ production when screening. To address these limitations, primary human thyrocytes, isolated from healthy adult donor tissues and cryopreserved at passage one (p’1) were characterized for cellular composition, 3D follicular architecture, and thyroglobulin (TG)/T_4_ expression and inhibition by prototype thyroid disrupting chemicals (TDC). Flow analysis of the post-thaw cell suspension showed >80% EpCAM-positive cells with 10%–50% CD90-positive cells. When seeded onto 96-well Matrigel^®^-coated plates and treated with bovine thyroid stimulating hormone (TSH), thyrocytes formed 3D microtissues during the initial 4–5 days of culture. The microtissues exhibited a stable morphology and size over a 14-day culture period. TG and T_4_ production were highest in microtissues when the proportion of CD90-positive cells, seeding density and thyroid stimulating hormone concentrations were between 10%–30%, 6K–12K cells per well, and 0.03–1 mIU/mL, respectively. At maximal TG and T_4_ production levels, average microtissue diameters ranged between 50 and 200 µm. The T_4_ IC_50_ values for two prototype TPO inhibitors, 6-propyl-2-thiouracil and methimazole, were ∼0.7 µM and ∼0.5 µM, respectively, in microtissue cultures treated between days 9 and 14. Overall, p’1 cryopreserved primary human thyrocytes in 3D microtissue culture represent a promising new model system to prioritize potential TDC acting directly on the thyroid as part of a weight-of-evidence hazard characterization.

## Introduction

Thyroid hormones (TH) play an important role in regulating the normal growth, development, and energy metabolism of different organ systems. Approximately 30 million people in the United States are affected by hypothyroidism, one of the most common endocrine diseases worldwide. Primary hypothyroidism results when the thyroid produces insufficient TH, presenting as a decrease in thyroid hormone thyroxine (T_4_) and an increase in thyroid stimulating hormone (TSH) (Chaker et al., 2017; Chiovato et al., 2019). Hypothyroidism can have substantial clinical impacts, particularly during pregnancy, and early childhood, leading to adverse outcomes such as preeclampsia, preterm birth (Männistö et al., 2013), and congenital hypothyroidism, which impairs neurodevelopment in children (Komur et al., 2013; Ehsani et al., 2021). While iodine deficiency is considered the most prevalent etiology for primary hypothyroidism (Melse-Boonstra and Mackenzie, 2013; Ghanbari and Ghasemi, 2017), increasing epidemiological evidence demonstrates that environmental chemical exposure can significantly disrupt normal circulating TH levels (He et al., 2024).

In order to screen for potential chemicals that might pose the risk of endocrine disruption in humans and wildlife, the U.S. Environmental Protection Agency (EPA) has developed the Endocrine Disruptor Screening Program (EDSP). This two-tiered testing process includes the identification of compounds of interest and investigations of dose-response relationships between the compounds and the thyroid hormone systems (Environmental Protection Agency, 2022). Similarly, the European Chemical Agency and the European Food Safety Authority have jointly published guidance on endocrine disruptor identification, including an assessment strategy for determining the endocrine effects of substances (Andersson et al., 2018). This international focus on rapidly evaluating potential thyroid-disrupting compounds highlights the need for a high-throughput screening approach to replace or limit the use of the low-throughput, animal-dependent conventional approach which has become the current regulatory industry standard.

Recent efforts to implement *in vitro* strategies to evaluate potential adverse outcomes from environmental chemical exposure involve the use of microsomes or cell lines targeting key molecular components in thyroid biological pathways, including thyroperoxidase (TPO), sodium iodide symporter (NIS), and deiodinase type I (DIO I) enzyme inhibitors (Paul Friedman et al., 2016; Hallinger et al., 2017; Hornung et al., 2018). While these assays provide important tools for rapid screening of compounds to predict toxicity, they do not adequately recapitulate human thyroid cellular physiology and architecture and are therefore limited in terms of modeling the complex biochemical interactions of the thyroid gland. The synthesis of T_4_ and tri-iodothyronine (T_3_) depends on the formation of functional units called thyroid follicles which are comprised of a single outer layer of epithelial cells (thyrocytes) surrounding an internal lumen (colloid) where the synthetic steps of thyroid hormones take place (Rousset et al., 2000). This intact follicular architecture is critical for TH synthesis and secretion to occur at the tissue level (Pirahanchi et al., 2023). In addition, long term culture and multiple passages of thyrocytes or immortalized cell lines leads to the loss of their native properties and synthetic capabilities, including response to TSH and disturbed karyotypes (van Staveren et al., 2007). Immortalized thyroid cells capable of forming the 3D structure have been characterized, although these cells failed to secrete T_4_ following TSH induction (Hopperstad et al., 2021).

Recently, Deisenroth *et. al.* described a novel 3D culture platform for thyroid disrupting chemical (TDC) screening using reconstructed thyroid microtissues comprised of primary human thyroid-derived cells at passage 0 to 2 (Deisenroth et al., 2020; Foley et al., 2024). Under these specific culture conditions, the freshly isolated primary thyrocytes were able to restore their native structural features and capability to synthesize thyroid hormone. Building on this organotypic model, the aim of the current study was to investigate the cellular composition of cryopreserved human thyroid cells at passage one (p’1) and better define the culture and treatment conditions for optimal microtissue formation and performance for use as a model system for TDC testing. In this study, cryopreserved p’1 thyrocytes were characterized for purity and marker expression, confirmed by flow analysis of post-thaw cell suspensions using antibodies to EpCAM, CD90, and CD144, and by immunostaining of 2D cultures for Cytokeratin 7 (CK7) and Fibroblast Specific Protein 1 (FSP1). Using the 3D culture platform, microtissue formation was followed over a 2-week culture period, and levels of TG and T_4_ were measured. Optimized assay conditions for TG and T_4_ production were determined including cell seeding density, microtissue size, and TSH concentration. Finally, application of the optimized 3D culture was evaluated *via* exposure to thyroid-disrupting reference compounds, 6-propyl-2-thiouracil and methimazole. The results of this study demonstrate that 3D microtissues of cryopreserved p’1 primary human thyroid cells, when cultured under optimal conditions, represent a relevant model system for screening TDC for potential impact on the thyroid gland.

## Materials and methods

### Culture medium preparation

Modified h7H human thyrocyte culture medium (HTCM) was used for all thyrocyte culture experiments as reported previously (Foley et al., 2024). Medium was sterile filtered through a 0.22 µm PES filter unit (ThermoFisher Scientific, Waltham, MA).

### Thyrocyte cell preparation

All methods were performed in accordance with the guidelines and regulations of LifeNet Health’s ethics committee. Informed consent was obtained for all donor tissue for research purposes by LifeNet Health. Thyrocytes from adult euthyroid donor tissues were isolated using modifications of a previously published isolation and cryopreservation protocol (Deisenroth et al., 2020). A panel of donors, male and female, used in this study were of varying ethnicity, age, and body mass index (BMI) (Table 1).

**TABLE 1:** Identification of each donor lot number plus specifications including sex, age, race, and BMI are listed.

Donor	Sex	Age	Race	BMI
2020525	Male	22	Caucasian	29.1
2024220	Male	50	African American	21.6
2021159	Male	43	Caucasian	23.4
2021598	Male	43	African American	32
2120653	Male	55	Caucasian	24.5
2215425	Female	51	Caucasian	28
2214495	Male	22	Hispanic	27
2216507	Male	38	Caucasian	23.5
2217737	Female	27	Caucasian	33
2313606	Female	22	Caucasian	23
2318558	Male	18	African American	22
2318632	Female	45	Caucasian	23

### Thawing and cell culture (2D or 3D) of cryopreserved P′1 thyrocytes

For functionality studies, 96-well white-wall clear-bottom microplates were used (Perkin Elmer, Waltham WA). For immunostaining, 96-well black-wall clear-bottom microplates were used (Corning, CA). All microplates were tissue culture-treated and either coated with 50 µL of Matrigel^®^ at the concentration of 9–10 mg/mL for 3D culture or left uncoated for 2D applications. Coated microplates were incubated for 1 h at 37°C CO_2_ to allow Matrigel^®^ to solidify. Cryopreserved human thyrocytes were thawed in a 37°C water bath for ≥90 s until the cell suspension liquified and could slide freely when the vials are inverted (approx. 2 min max) before being added dropwise into 14 mL of human thyrocyte plating medium (HTPM), which is HTCM without bovine TSH. The cell suspension was centrifuged for 5 min at 200 x g in a swing bucket rotor. The pellet was resuspended in 1 mL of fresh plating medium, stained with 0.4% Trypan Blue (Gibco, Billings, MT) and counted using an automated cell counting system. Cells were further diluted with HTPM to the appropriate final volume, and 100 µL was added to each well excluding the outer wells at the final cell seeding densities specified in the Results section. Microplates were then incubated in a 37°C CO_2_ incubator. On day 2, cell culture medium was replaced with HTCM containing bovine TSH (Sigma-Aldrich, St. Louis, MO) at the concentration indicated for each data set and maintained in the same medium until day 14 with medium exchange on days 5, 7, 9, and 12.

### Hematoxylin and Eosin staining

For H&E staining, microtissues on day 12 were fixed with 10% formalin prior to processing and paraffin embedding. Blocks were mounted and 4-µm cross sections were cut, deparaffined, and stained with Gill 2 Hematoxylin and Eosin Y (Richard Allan Scientific, San Diego, CA) for 2 and 3 min respectively. Slides were dehydrated and imaged using a Ziess Observer Z1 microscope (Zeiss, Dublin, CA).

### Immunofluorescent staining

For CK7 and FSP1 staining, thyrocytes were seeded at 100,000 cells per chamber on an 8-well chamber slide (ThermoFisher Scientific). After 18–24 h, the medium was aspirated and 250 µL of Cytofix/Cytoperm solution (BD Biosciences, San Jose, CA) was added to each well followed by a 30 min incubation at 4°C for cell fixation. Samples were then washed 2 times with 250 µL of 1X Perm/Wash buffer (BD Biosciences). Primary antibodies were added including monoclonal mouse anti-human CK7 (Clone OV-TL, Agilent Dako, Santa Clara, CA) (1:100 dilution) and rabbit polyclonal FSP1 (Abcam, Boston, MA) (1:100 dilution), and incubated overnight at 4°C. After overnight incubation, samples were washed with 1X Perm/Wash buffer followed by the addition of Goat Anti-mouse Alexa 488 IgG (H + L) (1:500) (Life Technologies, Carlsbad, CA) or Goat anti-rabbit Alexa-555 IgG (H + L) (1:500) (ThermoFisher). The chambers were then incubated at room temperature for 1 h in the dark. Samples were washed with 1X Perm/Wash buffer and followed by a wash with 1X DPBS (ThermoFisher Scientific). Finally, two drops of Fluoromount G with DAPI (ThermoFisher Scientific) were added into each chamber prior to imaging. Images were acquired and processed using Ziess Observer Z1 fluorescent microscope with Apotome-2 and Zen Image Acquisition Software.

For actin microfilament staining, thyrocytes were seeded at 7,500 cells per well and cultured as 3D microtissues for 9 days. On day 9, microtissues were fixed with Cytofix/Cytoperm as described above, and Alexa Fluor 488-labeled Phalloidin (ThermoFisher Scientific) (1:200 dilution) was added. After a 2-h incubation period at room temperature in the dark, samples were washed and stained with 300 nM DAPI (ThermoFisher Scientific) for 1 h in the dark at room temperature. The cells were then washed with 1X DPBS, and Fluoromount G with DAPI was added to each sample. Images were acquired and processed as described above using ×100 magnification on the Ziess Observer Z1 fluorescent microscope.

### Enzyme-linked immunosorbent assays (ELISA)

Human TG and T_4_ detection and quantification were carried out using the EHTG and the EIAT4C ELISA kits (ThermoFisher Scientific). Assays were performed according to the manufacturer’s instructions using medium collected from cell culture wells at day 7 for TG and day 14 for T_4_ ELISA. A CLARIOStar (BMG Labtech) microplate reader was used for data collection. Medium collected for TG assay was diluted at a ratio of 1:200 for 2D cultures and 1:100 to 1:200 for 3D cultures. Medium collected for T_4_ assay was undiluted. MARS (version 3.31) software was used for data analysis according to the manufacturer’s manual.

### Flow cytometry

Anti-human Epithelial Cell Adhesion Molecule (EpCAM) (CD326, clone: VU-1D9)- FITC (Stem Cell Technologies, Vancouver, BC), anti-human CD90 (Thy-1, clone: 5E10)- Alexa Fluor 700 (ThermoFisher Scientific), and anti-human CD144 (clone: 55-7H1)- PerCPCy5.5 (BD Biosciences) were used for thyrocyte staining at 1:100 dilution. After viability and cell count measurements were determined post-thaw, cells were centrifuged at 250 x g for 5 min at room temperature, and the supernatant was discarded. Cell pellets were resuspended in 600 µL of BD Pharmingen Stain Buffer (BD Biosciences), and a 100 µL volume was aliquoted into labelled FACS tubes for unstained, FMO, and full stain samples appropriately. UltraComp eBeads^TM^ Plus Compensation beads (ThermoFisher Scientific) were prepared for single stain compensation controls. The bead vial was vortexed for 30 s and one drop was added into each labeled control FACS tube. Each tube was vortexed for 30 s then incubated for 30 min at 4°C in the dark. An Attune Nxt Flow Cytometer (ThermoFisher Scientific) was used for data acquisition, and *De Novo* Software–FCS Express 7 RUO Edition (7.12.0005) was used for data analysis. Between 5,000–10,000 events were recorded per single stain control, and ≥50,000 events were recorded per sample. Cells of interest were identified and gated based on forward side scatter area (FSC-A) against the side scatter area (SSC-A) from which single cells were gated using FSC-A and forward side scatter height (FSC-H). Cells positive for the markers of interest were identified based on the gating of each marker against SSC-A. Background fluorescence and gating boundaries were determined using unstained and FMO control tubes.

### Morphological assessment and measuring microtissue diameter

To morphologically assess the cells, they were imaged on the designated days using a BX41 microscope (Olympus, Tokyo, Japan). ImageJ was used to measure microtissue diameter using images collected using a ×4 objective on a BX41 microscope (Olympus). To ensure the accurate measurement of the microtissues, the scale bar on the image was measured through the software. The diameters of the microtissues were measured in µm from ≥5 images taken on days 1, 2, 5, 7, 9, 12, and 14. For each condition, a minimum of 10 microtissues were measured. The average diameter and standard deviation were calculated using Microsoft Excel.

### Reference chemical evaluation

The inhibitors, 6-propyl-2-thiouracil (CASRN 51–52-5) and Methimazole (CASRN 60–56-0), were obtained from MilliporeSigma (Burlington, MA). Thyrocytes were seeded on Matrigel^®^-coated plates at a density of 7,500 cells per well (0.32 cm^2^) to form microtissues and were treated with 1 mIU/mL bovine TSH on days 2, 5, 7, 9, and 12. For each studied compound, cells were dosed on days 9 and 12 with concentrations ranging from 0.0001 to 10 μM at log intervals. T_4_ and ATP levels were measured as described. All measured data were normalized to DMSO vehicle control.

### Cell titer Glo assay

In order to assess whether the reference chemicals posed any toxic effects to the thyrocytes, CellTiter-Glo assay was used to measure ATPase activity of samples on day 14 to determine cell viability. After collecting medium samples for T_4_ measurement, backing tape was put on the back of the 3D culture 96-well plate, and 100 µL volume of a 1:1 mixture of CellTiter-Glo^®^ Luminescent Cell Viability Assay (Promega, Madison, WI) and HTCM was added into each well of the 3D cell culture plate. The plate was covered and shaken for 2 min on an orbital plate shaker at 500 rpm followed by a 10 min incubation in the dark without shaking. Luminescence reading was recorded using a CLARIOStar (BMG Labtech) microplate reader.

### Statistical analysis

Images are shown from representative donor lots. Values were normalized to the number of cells seeded per well. Significance was calculated in MiniTab (State College, PA) using either Student’s t-test between two groups or One-way ANOVA with Tukey *post hoc* testing when comparing three groups or more to determine statistical significance with 95% confidence and *p* < 0.05 for statistical significance. GraphPad Prism v.9 (GraphPad Software, Boston, MA) was used to determine EC_50_ and EC_90_ values. Four-parameter nonlinear regression fit was used to determine the half-maximal inhibition concentration IC_50_ using GraphPad Prism v.9. Microsoft Excel was used to determine R^2^ values.

## Results

### 3D microtissue formation in culture

**FIGURE 1:**
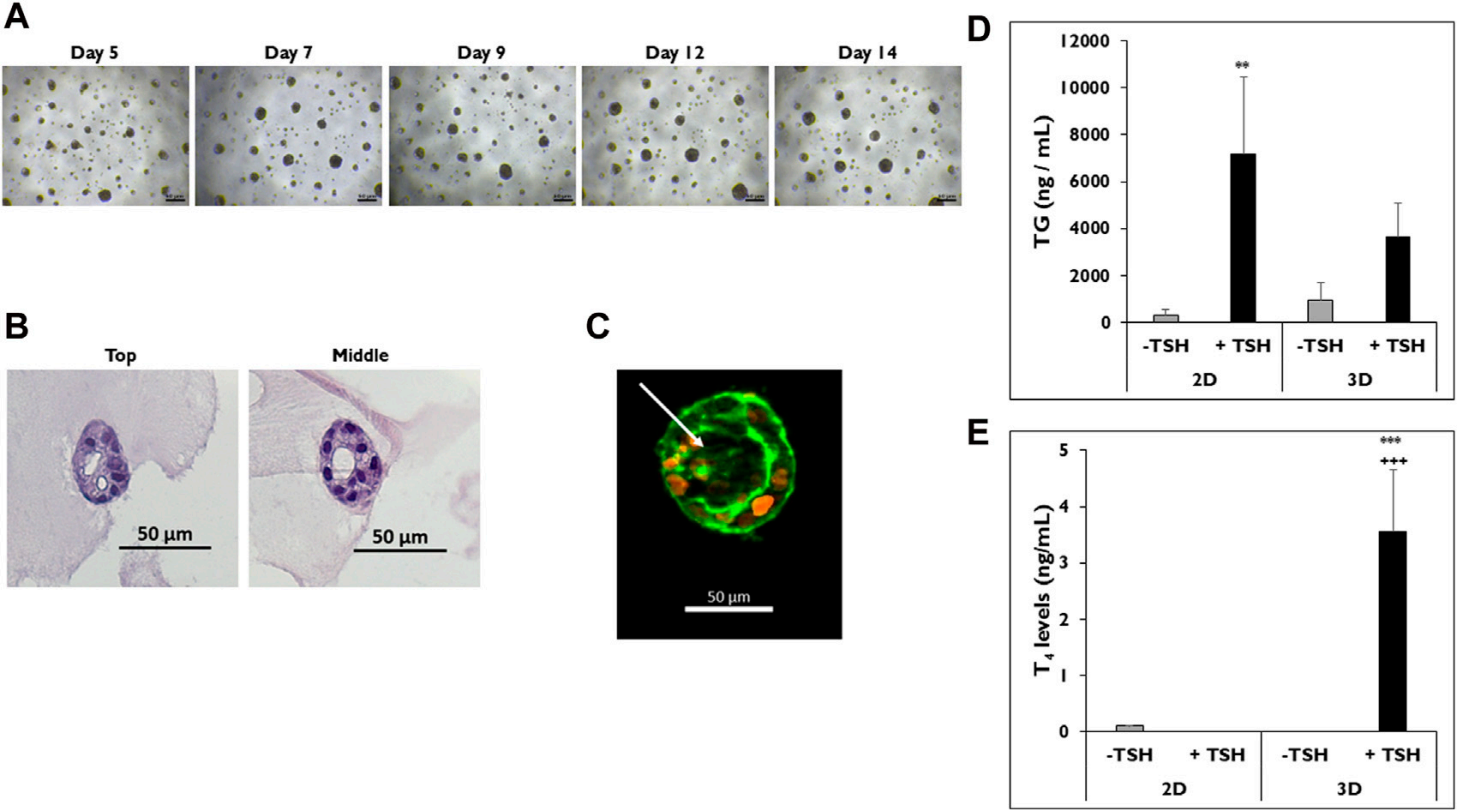
Thyrocytes in 3D culture form microtissues. **(A)** Representative images of microtissues on days 5, 7, 9, 12, and 14 in 3D culture. Magnification ×40. Scale bar = 50 µm. **(B)** H&E staining of microtissues, including follicle-like structures, from top to middle on day 12. Magnification ×200. Scale bar = 50 µm. **(C)** Follicle-like morphology of microtissue stained with DAPI (orange) and phalloidin (green) on day 9. Arrow indicates luminal space absent of cell staining. Magnification ×100. Scale bar = 50 µm. Representative data from one donor for levels of **(D)** thyroglobulin (TG) on day 7 and **(E)** Thyroxine (T_4_) on day 14 from thyrocytes in 2D and 3D culture treated without (grey bars) and with (black bars) TSH. 7,500 cells per well were seeded for each culture condition. Cells were treated with Thyroid Stimulating Hormone (1.0 mIU/mL). One-way ANOVA with Tukey for stats. Error bars represent standard deviation. n = 3 replicates from three donor lots. **p < 0.005 to–TSH 2D for TG. ***p ≤ 0.001 to -TSH 3D; +++ *p* ≤ 0.001 to + TSH 2D for T_4_.

Cryopreserved p’1 primary human thyrocytes were seeded onto Matrigel^®^-coated 96-well plates to determine if cells would self-aggregate and form microtissues that resemble their native follicle-like structure *in vivo* (Figure 1). Cells began to aggregate, starting 24 h after seeding, and distinct, stable individual microtissues formed by day 5 (Figure 1A). These microtissues had a smooth, round shape with clearly defined borders throughout the 14-day culture period. The morphology of these microtissues by day 7 showed distinct follicle-like structures with empty pockets absent of cells, which can be seen from the top and middle sections of H&E stained microtissues on day 12 (Figure 1B). Actin staining of these microtissues using fluorescently-labeled phalloidin showed a similar distribution of cells surrounding an inner lumen devoid of cells. The outer ring of cells exhibited a typical peripheral localization of microfilaments at the outer and inner membrane edges (Figure 1C).

Once microtissues formed, their functionality was measured by determining TG and T_4_ secretion. Cells cultured in 2D were used as a control to demonstrate sensitivity to TSH stimulation. Thyrocytes in both 2D and 3D cultures secreted TG when stimulated with TSH (Figure 1D). Cells in 2D culture treated with TSH secreted more TG compared to microtissues in 3D culture (7,181 ± 3,291 vs. 3,658 ± 1,437 ng/mL). Cells in 2D treated with TSH secreted significantly higher levels of TG compared to non-treated cells (327 ± 214 ng/mL). When the corresponding T_4_ levels were measured in the medium samples, microtissues in 3D culture treated with TSH secreted significantly higher levels of T_4_ compared to 2D cultures, which secreted little to no T_4_ with or without TSH stimulation (3.5 ± 1.1 vs*.* ND ng/mL) (Figure 1E).

### Characterization of distinct cell populations

**FIGURE 2:**
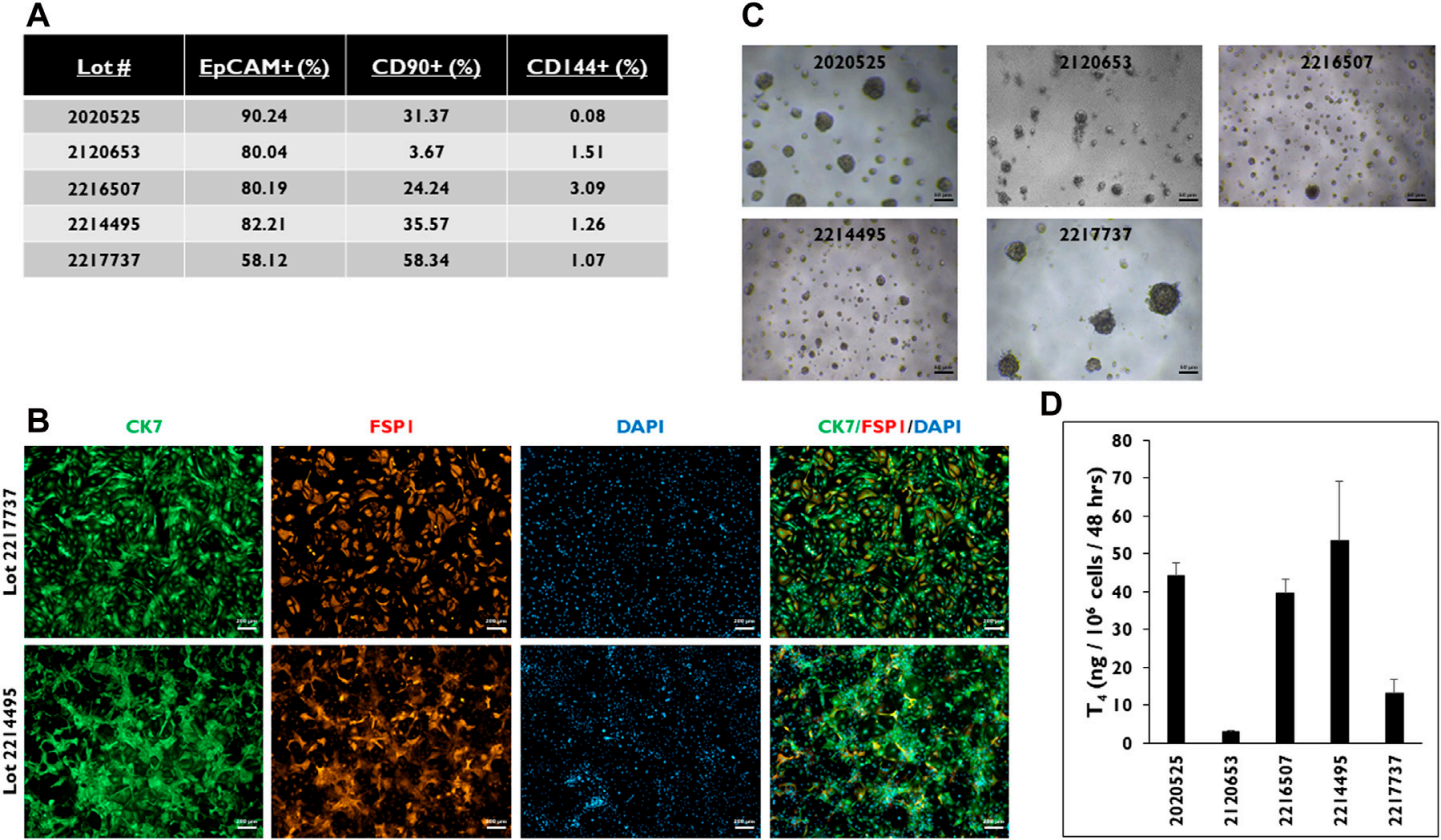
Characterization of the cell population from five donor lots determined by FLOW. **(A)** Percentage of cells stained positive for EpCAM, CD90, and CD144. > 0.5 × 10^6^ viable cells were used for each of the five donor lots. **(B)** Representative images of thyrocyte cells from donor lots 2217737 (top row) and 2214495 (bottom row) in 2D culture stained for Cytokeratin 7 (CK7, green), Fibroblast-specific protein 1 (FSP1, red), and DAPI (blue), and three channels merged. Magnification ×50. Scale bar = 200 µm. **(C)** Representative images of microtissues in 3D culture seeded at 7,500 cells per well and **(D)** levels of Thyroxine (T_4_) from microtissues in 3D culture on day 14. Cells were treated with Thyroid Stimulating Hormone (1.0 mIU/mL). Magnification ×40. Scale bar = 50 µm. Error bars represent standard deviation. n ≥ 3 replicates from five donor lots.

Because the formation of 3D microtissues involves the aggregation of primary cells, it was important to determine the types of cells forming them (Figure 2). Flow cytometry was performed on five donor lots to characterize which cell types are present when these cells are seeded in 3D culture (Figure 2A). Three markers of cell lineage were used, including EpCAM, CD90, and CD144 for epithelial, stromal, and endothelial cell lineages, respectively. Four of the five donor lots had ≥80% EpCAM-positive cells. The fifth donor lot, 2217737, had ∼58% EpCAM-positive cells. This donor lot also had the highest number of CD90-positive cells (∼58%). Donor lot 2120653 had the lowest number of CD90-positive cells (∼3.7%). All five donor lots had <5% CD144-positive cells. Immunostaining for CK7 and FSP1, markers of epithelial and stromal cell lineages, was performed on 2D cultures of cells isolated from donor lots 2217737 and 2214495 (Figure 2B). Both donor lots stained positive for CK7, while donor lot 2217737 appeared to have a distinctly higher pattern of FSP1-positive cells compared to donor lot 2214495.

When cells from these donor lots were seeded in 3D culture, each of the lots had microtissues of varying sizes and numbers (Figure 2C). For example, cells from donor lot 2120653 appeared to form the smallest microtissues. Cells from donor lot 2217737 appeared to have the fewest number of microtissues per well compared to the other four donor lots tested along with microtissue size visually comparable to lot 2020525. When the microtissues were stimulated with TSH, cells from donor lots 2120653 and 2217737 secreted the lowest levels of T_4_ compared to cells from the other donor lots (3.2 ± 0.3 and 13.2 ± 3.7 ng/10^6^ cells/48 h) (Figure 2D). The T_4_ levels from the other donor lots ranged from 53.6 to 39.6 ng/10^6^ cells/48 h.

### Effect of seeding density on microtissue formation in 3D culture

**FIGURE 3:**
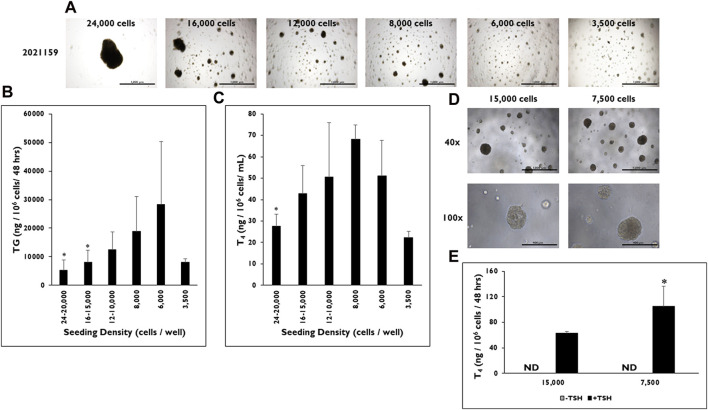
The effect of seeding density range on microtissue formation and function in 3D culture. **(A)** Representative images of microtissue formation on day 14 when 24,000, 16,000, 12,000, 8,000, 6,000, and 3,500 cells are seeded per well. Magnification ×40. Scale bar = 1,000 µm. **(B)** Thyroglobulin (TG) and **(C)** Thyroxine (T_4_) levels were determined at various seeding density ranges from 24 to 20,000 cells per well to 3,500 cells per well. Values were normalized to the number of cells seeded per well. *p < 0.05 to 6,000 cells per well for TG (One-way ANOVA with Tukey). *p < 0.05 to 8,000 cells per well for T_4_ (One-way ANOVA with Tukey). n ≥ 2 replicates from three donor lots for TG and two donor lots for T_4_ analysis. **(D)** Representative images of microtissues on day 9 from donor lot 2313606 formed when cells were seeded at 15,000 and 7,500 cells per well. Magnification at ×40 (top row) and ×100 (bottom row). Scale bar = 1,000 μm and 400 µm. **(E)** T_4_ levels on day 14 were measured from microtissues (donor lot 2313606) formed when cells were seeded at 15,000 cells per well and 7,500 cells per well. Cells were treated with Thyroid Stimulating Hormone (0.3 mIU/mL). *p < 0.05 to 15,000 cells per well + TSH. One-way ANOVA with Tukey for stats. n = 5 replicates from one donor lot. Error bars represent standard deviation.

Microtissue formation in 3D culture is a necessary component for T_4_ secretion; therefore, the effect of the number of cells seeded per well on microtissue formation was examined (Figure 3). Cells were seeded at different densities from 24–20,000, 16–15,000, 12–10,000, 8,000, 6,000, and 3,500 cells per well (Figure 3A). Microtissues of varying sizes and shapes formed at the different seeding densities. At the highest seeding density (24–20,000 cells per well), one to two large, non-symmetrical microtissues formed per well, which visually appeared to be much larger than the other microtissues seeded at the lower seeding densities. Microtissues appeared to decrease in size as the seeding density was lowered; however, cells were still able to self-organize into microtissues at the lowest seeding density of 3,500 cells per well in 3D culture.

TG and T_4_ production rates were measured from microtissues in 3D culture formed at each seeding density. As the seeding density decreased, the rate of TG production increased from microtissues stimulated with TSH (Figure 3B). However, there was a drop off in the TG production rate (8,079 ± 1,125 ng/10^6^ cells/48 h) at the lowest seeding density of 3,500 cells per well. Cells that were seeded at 6,000 cells per well formed microtissues that secreted the highest level of TG (28,399 ± 21,870 ng/10^6^ cells/48 h). Significantly lower TG levels were measured from microtissues formed from cells seeded at the highest seeding densities of 24–20,000 (5,283 ± 3,538 ng/10^6^ cells/48 h) and 16–15,000 cells per well (8,151 ± 4,224 ng/10^6^ cells/48 h) compared to microtissues seeded at 6,000 cells per well.

A similar trend was seen for T_4_ production rates at the different seeding densities (Figure 3C). As the number of cells seeded per well decreased, the rate of T_4_ production increased until reaching a maximum at 8,000 cells per well (68.0 ± 6.8 ng/10^6^ cells/48 h). However, a drop off in secretion was seen from microtissues formed at the 6,000 and 3,500 cells per well seeding densities (51.1 ± 16.4 ng/10^6^ cells/48 h and 22.2 ± 3.0 ng/10^6^cells/48 h). Likewise, T_4_ production levels were significantly lower from microtissues formed at 24–20,000 cells per well (27.6 ± 5.7 ng/10^6^ cells/48 h) compared to the 8,000 cells per well seeding density. The lowest rate of T_4_ production was from microtissues seeded at 3,500 cells per well (22.2 ± 2.9 ng/10^6^ cells/48 h).

As further confirmation of the optimized seeding density, two seeding densities from donor lot 2313606 were tested at seeding densities of 15,000 and 7,500 cells per well. Both seeding densities formed microtissues that visually looked similar in 3D culture (Figure 3D). However, the microtissues formed from cells seeded at the 7,500 cells per well had significantly higher rates of T_4_ production compared to the microtissues formed from cells seeded at 15,000 cells per well when stimulated with TSH (105.2 ± 31.28 vs. 62.6 ± 2.8 ng/10^6^ cells/48 h, respectively) after normalizing to seeded cell number (Figure 3E).

### TSH stimulation and differences in microtissue diameter in 3D culture

**FIGURE 4:**
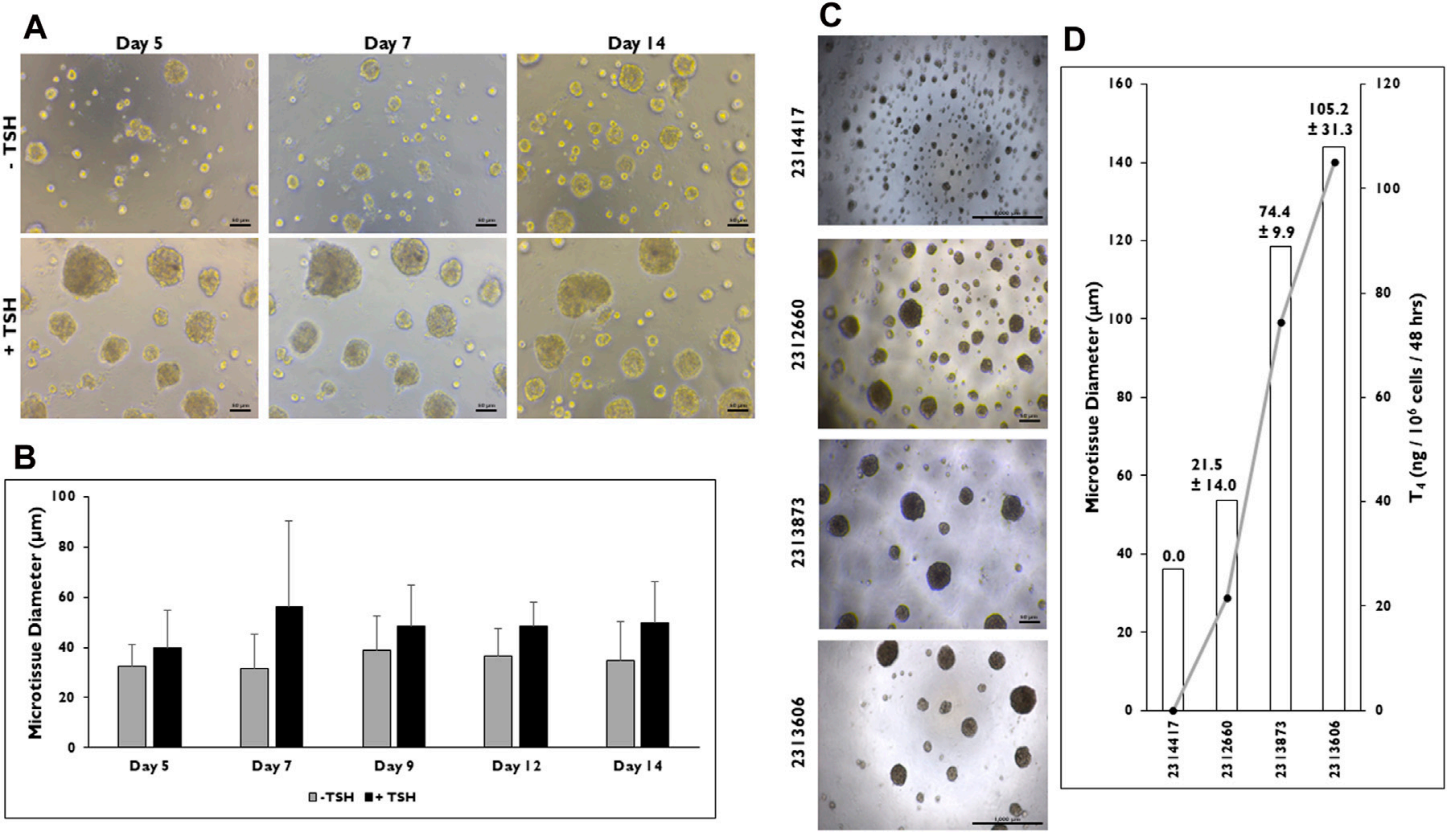
Microtissue size can be correlated to T_4_ synthesis in 3D culture. **(A)** Representative images of microtissues on days 5, 7, and 14 that have been treated without (−) or with (+) Thyroid Stimulating Hormone (TSH) (0.3 mIU/mL) seeded at 7,500 cells per well beginning at day 2. Magnification ×100. Scale bar = 50 µm. **(B)** Measurements of microtissue diameter (µm) on days 5, 7, 9, 12, and 14 without (grey bars) and with (black bars) TSH treatment. n = 10 microtissues from one donor lot. **(C)** Representative images of microtissues on day 12 from four donor lots when cells were seeded at 7,500 cells per well and treated with TSH (0.3 mIU/mL). Magnification ×40. Scale bar = 1,000 µm for donor lots 2314417 and 2313606. Scale bar = 50 µm for lots 2312660 and 2313873. **(D)** Levels of T_4_ synthesis (grey line) from four donor lots correlated to microtissue diameter (µm) on day 14. T_4_ values were normalized to number of cells seeded per well. n ≥ 10 microtissues measured and n = 5 replicates from four donor lots for T_4_ levels. Errors bars represent standard deviation.

Relative differences in size were observed between non-stimulated *versus* TSH stimulated microtissues in 3D culture (Figure 4). Representative images of microtissues treated without or with TSH on days 5, 7, and 14 are shown (Figure 4A). The results showed that larger microtissues are present when stimulated with TSH. To confirm this, microtissue diameter was determined between the two groups on days 5, 7, 9, 12, and 14 (Figure 4B). Larger microtissues on average were measured starting on day 5 (39.8 ± 14.8 vs. 32.4 ± 8.8 µm), peaking by day 7 (56.0 ± 34.5 vs. 31.7 ± 13.4 µm), and remaining until day 14 (49.6 ± 16.5 vs. 34.6 ± 15.6 µm) when treated with TSH *versus* non-stimulated. Overall, the average range of diameters is consistently larger in the TSH stimulated microtissues.

Because microtissues increase in diameter due to TSH stimulation, the relationship between microtissue size and T_4_ production rate was determined in microtissues from four donor lots seeded at the same density (7,500 cells per well) (Figure 4C). There were visual differences in microtissues between each donor lot. Donor lot 2314417 appeared to form the smallest microtissues, while donor lot 2313606 appeared to form the largest. When microtissue diameter was measured, donor lots 2314417 and 2312660 formed microtissues averaging 36.1 ± 3.7 and 53.8 ± 9.7 µm in diameter (Figure 4D), while larger microtissues were measured from donor lots 2313873 and 2313606 (118.7 ± 36.7 and 143.9 ± 42.5 µm).

T_4_ production rate was found to be correlated to microtissue size as microtissue diameter increased, T_4_ levels increased proportionally (Supplementary Figure S1A). No detectable quantity of T_4_ was measured from donor lot 2314417 where average microtissue diameter was <40 µm. The remaining donor lots all secreted T_4_ with donor lot 2312660 having the lowest level (21.5 ± 14.0 ng/10^6^ cells/48 h). Donor lots 2313873 and 2313606 secreted the highest T_4_ levels (74.4 ± 9.9 and 105.2 ± 31.3 ng/10^6^ cells/48 h).

### TSH concentration-response profiles for TG and T_4_ synthesis

**FIGURE 5:**
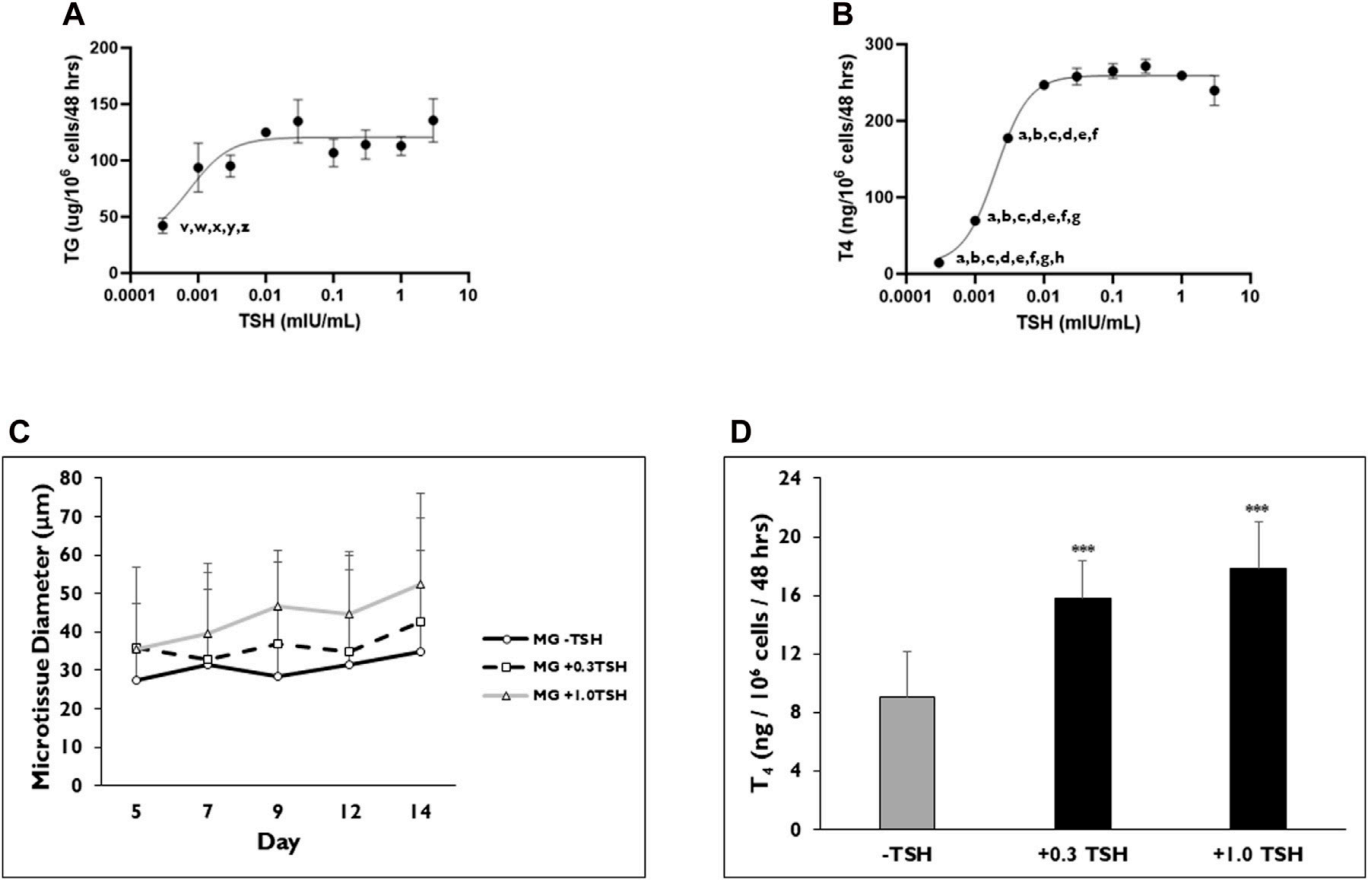
TSH concentration effects on TG and T_4_ synthesis from thyrocyte microtissues in 3D culture. Representative levels of **(A)** Thyroglobulin (TG) on day 7 and **(B)** Thyroxine (T_4_) on day 14 were measured when microtissues in 3D culture were stimulated with varying concentrations of Thyroid Stimulating Hormone (TSH) ranging from 0.0003 to 3 mIU/mL. Non-stimulated microtissues (0 TSH) secreted measurable levels of TG (31.7 ± 9.5 µg/10^6^ cells/48 h). Non-stimulated microtissues (0 TSH) produced T_4_ (17.63 ± 1 ng/10^6^ cells/48 h). *p* < 0.05 to 0.01 mIU/mL (indicated by z), 0.3 mIU/mL (indicated by x) and 1 mIU/mL (indicated by w) for TG. *p* < 0.01–0.03 mIU/mL (indicated by y) and 3 mIU/mL (indicated by v) for TG. *p* < 0.01 to 0.001 mIU/mL (indicated by h) for T_4_. *p* < 0.001–0.003 mIU/mL (indicated by g), 0.01 mIU/mL (indicated by f), 0.03 mIU/mL (indicated by e), 0.1 mIU/mL (indicated by d), 0.3 mIU/mL (indicated by c), 1 mIU/mL (indicated by b), 3 mIU/mL (indicated by a) for T_4_. One way ANOVA with Tukey. n = 3 replicates from two donor lots. Values were normalized to 7,500 cells per well. **(C)** Microtissue diameter was determined on days 5, 7, 9, 12, and 14 for cells seeded in Matrigel® (MG) after treatment without (black line, circles) or with 0.3 (dotted line, squares) and 1.0 (grey line, triangles) mIU/mL TSH beginning on day 2. n ≥ 5 images from one donor lot. **(D)** Levels of T_4_ synthesized from microtissues when treated with 0.3 and 1.0 mIU/mL TSH on day 14. ***p < 0.001 to–TSH. n ≥ 5 replicates from one donor lot. Errors bars represent standard deviation.

TG and T_4_ synthesis from microtissues in 3D culture are dependent upon TSH stimulation, but full concentration-response profiles have not been determined in this model system previously. The sensitivity of the microtissues to TSH concentration was determined on both TG and T_4_ production rates (Figure 5). For all the data sets, the cell seeding density was 7,500 cells per well. TSH concentrations were tested in half-log intervals between 0.0003 and 3 mIU/mL. There were no significant differences found in TG production when microtissues were stimulated between 0.001 mIU/mL (93.8 ± 37.7 µg/10^6^ cells/mL) to 3 mIU/mL (135.8 ± 33.2 µg/10^6^ cells/mL) TSH (Figure 5A). Significantly lower TG production was measured when microtissues were stimulated with 0.0003 mIU/mL TSH (42.2 ± 11.7 µg/10^6^ cells/mL) compared to the 0.01, 0.03, 0.3, 1, and 3 mIU/mL concentration. The EC_50_ and EC_90_ for TG secretion was 0.0008 and 0.003 mIU/mL, respectively. Non-stimulated microtissues (0 mIU/mL) secreted measurable levels of TG (31.7 ± 9.5 µg/10^6^ cells/48 h) that were not significantly different compared to the 0.0003 mIU/mL stimulated levels.

A concentration-dependent increase in T_4_ production was observed between TSH concentrations 0.003 and 0.01 mIU/mL (Figure 5B). No significant differences in T_4_ levels were measured from microtissues stimulated with TSH between 0.01 and 3 mIU/mL (247.09 ± 2 to 239.75 ± 39 ng/10^6^ cells/48 h). TSH concentration 0.3 mIU/mL (271.71 ± 19 ng/10^6^ cells/48 h) stimulated the highest T_4_ levels. The EC_50_ and EC_90_ for T_4_ secretion was 0.002 and 0.007 mIU/mL, respectively. Microtissues stimulated with TSH concentrations between 0.0003 (14.68 ± 2 ng/10^6^ cells/48 h) and 0.003 (177.73 ± 11 ng/10^6^ cells/48 h) mIU/mL secreted significantly less T_4_ compared to microtissues treated with the higher TSH concentration (0.01, 0.03, 0.1, 0.3, 1 and 3 mIU/mL). Although non-stimulated microtissues produced T_4_ (17.63 ± 1 ng/10^6^ cells/48 h), the level was not significantly different compared to those measured for the 0.0003 mIU/mL TSH stimulated microtissues.

Data presented previously showed that microtissue diameter increased with TSH stimulation, and T_4_ production rates were correlated with a minimum diameter of >40 µm for T_4_ synthesis. Higher concentrations of TSH were tested to determine the influence on microtissue size and corresponding T_4_ synthesis (Figure 5C). Microtissues were stimulated with either 0.3 or 1 mIU/mL TSH starting on day 2 of culture, and their average diameters were determined on days 5, 7, 9, 12, and 14. Both TSH concentrations caused microtissues to increase in diameter throughout the 14-day culture period. On day 7, microtissues had an average diameter of 32.9 ± 18.2 and 39.7 ± 26.4 µm on day 7 with 0.3 and 1.0 mIU/mL TSH stimulation, respectively. By day 14, TSH 0.3 and 1.0 mIU/mL stimulated microtissues increased in diameter to 42.7 ± 18.4 and 52.4 ± 41.1 µm. Non-stimulated microtissues ranged in diameter from 27.6 ± 11.6 to 35.0 ± 17.3 µm on days 5–14. When T_4_ production rates were measured, there was no significant difference in values between 0.3 and 1.0 mIU/mL TSH stimulated microtissues (15.8 ± 2.6 and 17.8 ± 3.2 ng/10^6^ cells/48 h, respectively), which were significantly higher compared to the levels from non-stimulated microtissues (9.0 ± 3.1 ng/10^6^ cells/48 h) (Figure 5D).

### Evaluation of thyroid-disrupting reference compounds

**FIGURE 6:**
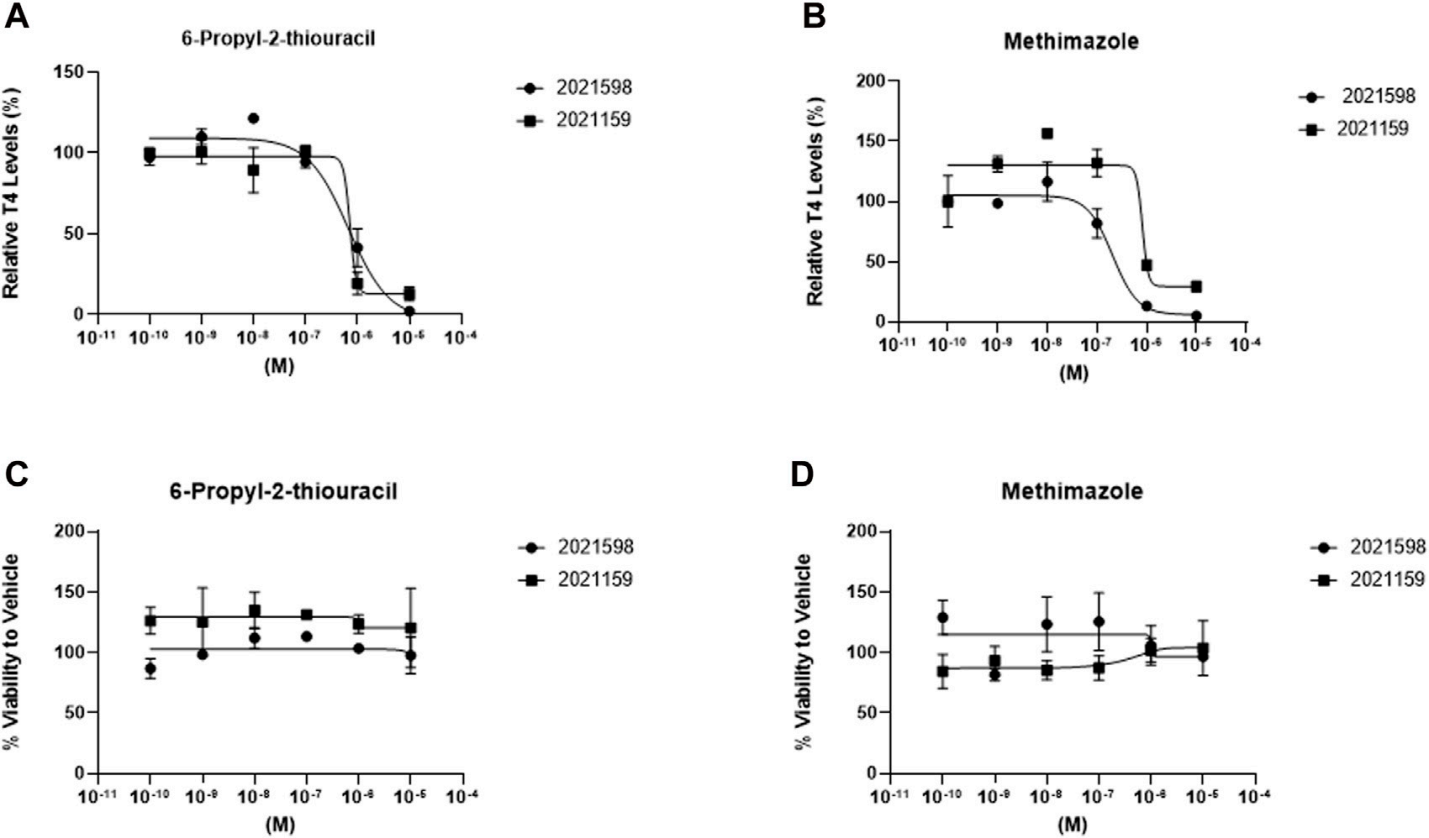
T_4_ synthesis from thyrocyte microtissues in 3D culture is inhibited by prototype thyroid disrupting compounds. Microtissues stimulated with 1 mIU/mL TSH on days 2, 5,7, 9, and 12 were treated with **(A)** 6-propyl-2-thiouracil and **(B)** methimazole using a dose response (1.0 × 10^−10^ to 1.0 × 10^−5^ M) between days 9 and 14 for donor lots 2021598 (circles) and 2021159 (squares). Viability for each donor was determined and normalized to Vehicle control exposed to 1mIU/mL TSH for treatment with **(C)** 6-propyl-2-thiouracil and **(D)** methimazole. n ≥ 3 replicates from two donor lots. Errors bars represent standard deviation.

Established disruptors of thyroid hormone production were selected to determine the concentration-response profiles in 3D cultures for TDC screening purposes (Figure 6). Inhibition of T_4_ synthesis was determined after a 120-h treatment between days 9 and 14 with 6-propyl-2-thiouracil, a TPO and DIO-1 inhibitor (Figure 6A), and methimazole, a TPO inhibitor (Figure 6B), at log concentration intervals between 1.0 × 10^−10^ to 1.0 × 10^−5^ M. No significant difference in viability was observed for either inhibitor (1.0 × 10^−10^ to 1.0 × 10^−5^ M) for donor lots 2021159 and 2021598. The concentration of both inhibitors was not toxic to the cells at the highest concentration of 1 × 10^−5^ M (Figures 6C,D). Average half-maximal inhibitory concentrations (IC_50_) for 6-propyl-2-thiouracil and methimazole were 6.6 × 10^−7^ M and 7.7 × 10^−7^, 2.2 × 10^−7^ and 7.9 × 10^−7^ M, respectively (Table 2). No differences in microtissue size were observed between treatment groups and vehicle control (data not shown).

**TABLE 2:** IC_50_ values for T_4_ inhibition were determined by a 4-parameter Hill model for TPO inhibitors, 6-propyl-2-thiouracil and methimazole.

Compounds	IC_50_ (M)	
	Donor 2021598	Donor 20211159
6-Propyl-2-thiouracil	6.6 × 10^−7^	7.7 × 10^−7^
Methimazole	2.2 × 10^−7^	7.9 × 10^−7^

## Discussion

The aim of this work was 1) to characterize cryopreserved primary human thyroid cells at passage one and 2) to investigate the culture and treatment conditions that best support the formation and maintenance of 3D microtissues for TDC screening applications. Determining the lineage of the cell types involved in 3D microtissue formation was performed using flow cytometry. Assessment of the expression of the epithelial marker EpCAM, the fibroblast marker CD90, and the endothelial marker CD144 post-thaw resulted in four out of the five donor lots tested having ≥80% EpCAM-positive cells. The percentage of CD90-positive cells in the five donor lots varied with three out of the five donor lots having ∼30% CD90-positive cells, and one donor lot having <5% and another donor lot having >50%. All five donor lots had ≤5% CD144-positive cells, indicating very little endothelial cell contribution to the final cell composition. Cells of endothelial origin (CD144-positive) averaged between 1% and 3% for four out of the five donor lots, while one donor lot was <0.1% (lot 2020525). Interestingly, when these donor lots were stimulated with TSH, T_4_ production levels varied. The donor lot that produced the lowest level of T_4_ had 80% EpCAM-positive cells but <5% CD90-positive cells (lot 2021653). The second lowest T_4_ producing donor lot had 58% EpCAM-positive cells and 58% CD90-positive cells (lot 2217737).

Based on these results, there appears to be a dynamic ratio of EpCAM-positive and CD90-positive cells that are needed for optimal formation of microtissue follicular architecture and the levels of thyroid hormone secreted into the medium. The overall flow data indicate that a relatively high number of EpCAM-positive cells with a mix of CD90-positive cells are minimally required at approximately a 4:1 or 3:1 ratio. The data also would suggest that these CD90-positive cells represent a mixed population of dual- and single-labeled cells; therefore, one distinct population that expresses only EpCAM and another distinct population that expresses both EpCAM and CD90. This is likely due to the presence of mature stromal cells and epithelial cells undergoing an epithelial-mesenchymal transition (EMT), likely during the initial expansion period (Kalluri and Weinberg, 2009). Regardless, the role of stromal cells in the deposition of extracellular matrix (ECM) and the development of tissue architecture likely contributes to the formation, diameter, and stability of the microtissue structure (Kendall and Feghali-Bostwick, 2014). A low number of CD90-positive cells might explain the lower T_4_ production rates observed in donor lot 2120653, despite appearing to form microtissues of suitable sizes. The high number of CD90-positive cells present in donor lot 2217737 could also account for the size of the microtissues with less effective T_4_ production. Although there appears to be a linear relationship between microtissue size and T_4_ production, the cell population must also be suitable for successful T_4_ production. Further studies are underway to explore the relationship between the proportions of epithelial and stromal cells and how this influences 3D microtissue structure and function.

The relationship between cell seeding density, microtissue formation, and T_4_ production was also determined. A similar trend of increasing TG and T_4_ synthesis rate in 3D microtissues when the cell seeding density was incrementally lowered from 20K to 8K cells per well was seen. However, distinctly different optimums for TG and T_4_ production rates were observed at a seeding density of 6K cells per well and 8K cells per well, respectively. Additional lots were tested to confirm this optimal seeding density range. Our hypothesis is that this may be due to the number of microtissues that can achieve a certain critical size range that closely mimics average follicular diameters observed in the thyroid gland *in vivo* (25–250 µm diameter) (Jackson, 1931). Confocal microscopic observations suggest that follicular-like structural features including luminal spaces surrounded by a single-layer of polarized epithelial cells of physiologically-relevant size and configuration form preferentially at specific cell seeding densities where maximal TG and T_4_ synthesis and secretion rates occur. These studies suggest that this optimally occurs in microtissues between ≥40 µm and ≤200 µm in diameter. However, the higher limit of the microtissue size was less limiting because even the largest microtissues formed secreted quantifiable amounts of T_4_. These findings agree with the findings of Faggiano et al. (2004) who reported that follicles in the small and intermediate size range (30–200 µm) were observed to be more active than larger follicles (≥200 µm), which were regarded as hypofunctioning. Moreover, these results would indicate that the preferred cell density to achieve this size range is between 6K and 12K cells per well. When cells are seeded using more than 12K cells per well or less than 6K cell per well, there is a decrease in T_4_ production. However, when cells are seeded within this range, there is no significant difference in T_4_ levels. Lastly, microtissue size seemed to stabilize on day 7 and remain constant for the rest of the culture period. Microtissue size from an additional donor was measured and showed a peak in size on day 7 with no significant changes during the remaining culture period. Further studies are warranted to better understand the relationship between cell seeding density, the size distribution and number of microtissues per well, and TG/T_4_ production efficiency.

The greatest source of variation in thyroid hormone production is donor-to-donor variance. Demographic characteristics, genetics, medical history, physiological and environmental factors are the essential elements of biological variability. Run-to-run consistency within the same lot of thyrocytes can be minimized by tight adherence to cell counting method, seeding density, and feeding regimens between runs. As demonstrated herein, microtissue size was positively correlated with thyroid hormone production. Future investigations will include strategies aimed at experimentally controlling microtissue size to determine if a more uniform, size-limited culture of microtissues reduces run-to-run and/or lot-to-lot variability.

The TSH concentration-response profile for TG and T_4_ production were determined in 3D p’1 microtissue cultures across a wide range of TSH concentrations (0.0003 mIU–3 mIU/mL). Non-TSH stimulated microtissues were tested for each donor lot to ensure significant production of T_4_. A standard TSH concentration used to stimulate cells *in vitro* is typically 1 mIU/mL, while circulating TSH levels for healthy adults (>20 years old) are between 0.5 and 5.0 mIU/mL (Biondi, 2013; Deisenroth et al., 2020). Results from 3D microtissue cultures that were continuously treated with a broad range of TSH concentrations between days 2 and 14 showed differential sensitivities in TG and T_4_ production. The lowest TSH concentration observed to stimulate TG production was 0.001 mIU/mL (or 1.0 µIU/mL) and remained stable up to the highest concentration of 3 mIU/mL. Stable T_4_ production started at 0.01 mIU/mL (or 10 µIU/mL) and remained consistent up to 3 mIU/mL. In addition to the use of 1 mIU/mL TSH to stimulate cells, decreasing the concentration to 0.3 mIU/mL TSH to stimulate cells can also be used. This concentration is ideally within the optimal TSH range for stimulation of TG and T_4_ while not being on the lower end of the range which may not be enough stimulation to measure a response for future applications. Additional donor lots have been tested to further confirm the TSH concentration-response profile. Furthermore, these studies utilized bovine TSH, which binds to human TSH receptor (hTSHR) with a higher affinity than human TSH and has 6–10-fold greater potency than human TSH (Mueller et al., 2009). Stimulation of the thyrocytes with bovine TSH may result in higher T_4_ synthesis in 3D model than physiological levels because of the greater affinity of the hormone to its receptor.

Synthesis of TG may be more sensitive to TSH due to its production being directly stimulated by TSH binding to the Thyroid Stimulating Hormone Receptor (TSHR) located on the follicle (Citterio, et al., 2021). However, T_4_ production is dependent upon multiple factors, including NIS/pendrin transporter functions, TPO activity, TG organization in the colloid spaces, and production of T_3_. These differences in TG and T_4_ production may also be reflected in the different culture conditions of 2D *versus* 3D. There was not a significant increase in TG production in the 3D culture that was determined in the 2D culture, which further suggests differences in regulation. Although TG levels influence synthesis of T_4_ *in vivo*, no correlation was found between levels of TG and T_4_ in these 3D microtissues (Supplementary Figure S1B). Because of the more complex production pathway of T_4_ and its dependence on multiple factors, higher concentrations of TSH may be required to see differences in stimulation. Future studies are needed to determine the minimum TSH concentration for stimulation of T_3_. The question remains whether T_3_ synthesis will have a similar profile to T_4_ or whether it could be somewhere in-between the sensitivities to TG and T_4_. Overall, the results from these experiments would suggest that optimal TSH concentrations for TDC screening are between 0.01 and 3 mIU/mL.

As a final step of the validation process, p’1 microtissue cultures were treated with known thyroid disrupting agents, methimazole (a potent TPO inhibitor) and 6-propyl-2-thiouracil (a TPO and DIO-1 inhibitor) at concentrations between 1.0 × 10^−10^ to 1.0 × 10^−5^ M. The efficacy and potency of their effects on T_4_ production rates were determined. The results showed a consistent concentration-dependent decrease in T_4_ levels for both inhibitors in two separate donor lots. The calculated IC_50_ values for each compound were consistent with those published in the human 3D thyroid microtissue assay and other published *in vitro* data using the microsomal assay (Deisenroth et al., 2020). Both methimazole and 6-propyl-2-thiouracil caused complete inhibition of T_4_ production at concentrations ≥10 mM. Further validation of the 3D microtissue assay using a broader range of selective and mixed-function pathway inhibitors and employing different endpoint assays to monitor shifts in key substrate levels (e.g., iodide uptake vs. T_4_ production rates) is currently underway. Methimazole and 6-propyl-2-thiouracil are potent TPO inhibitors that disrupt iodination of tyrosine residues and coupling reaction in thyrocytes. 6-propyl-2-thiouracil and methimazole are also antithyroid drugs (ATDs) and used to treat patients diagnosed with hyperthyroidism. Ochi et. al. (2018) reported that ATDs reduce the disulfide binds at the TSH-binding site of TSHR and enable the site for TSH and TSHR specific antibodies. High affinity of bovine TSH to hTSHR may interfere in mechanism of action for methimazole and 6-propyl-2-thiouracil which may result in higher effective concentrations of antagonist compounds.

The results from this study provide additional evidence for the potential contribution of this p’1 thyroid cell microtissue assay system for the testing of thyroid disrupting chemicals, especially for human risk assessment (Deisenroth et al., 2020; Foley et al., 2024). Historically, there has been a lack of primary cell-based model systems for human thyroid, especially models that exhibit the key features required for a more holistic approach to chemical testing, such as the physiologically relevant cellular architecture of the basic follicular substructure and the full complement of molecular and biochemical pathways pertinent to most of the key events established for the current thyroid adverse outcome pathway network. Unlike other human *in vitro* model systems currently available, such as microsomes and cell lines that express a limited subset of pathways, this 3D microtissue system could potentially be used to assess the more complex interactions that can occur simultaneously on multiple biochemical and molecular pathways (e.g., TSHR signaling, NIS transport, TPO/DIO inhibition). As there are multiple molecular initiating events (MIEs) in the thyroid axis that may result in adverse effects (Noyes et al., 2019) this 3D thyroid microtissue model will be useful to identify TDCs acting directly on thyroid and to evaluate multiple MIEs (e.g., TPO, NIS, TSHR, MCT8, pendrin) simultaneously. The 3D model will also confirm TDCs identified with high-throughput screening assays, allowing further testing a broader repertoire of hormone synthesis modulators. In addition, there is an opportunity to establish a new approach model and testing strategy for examining perturbations of the cellular architecture surrounding and within the colloidal space, which is at the heart of thyroid hormone production. Moreover, this system could help establish a ‘gold standard’ for determining the net effects of compounds on overall thyroid functions. For example, compound potency and efficacy data generated using single-pathway *in vitro* model systems, such as microsomes, could be put into better context relative to the overall contribution of that mode of action in the broader thyroid hormone production network by comparing the corresponding potency and efficacy data from the 3D microtissue assay.

In conclusion, this work provides further definition, characterization, and optimization of the first commercially available cryopreserved primary human thyroid cells. The results from this study show that, when cultured on Matrigel^®^-coated plates, the cells form stable 3D microtissues that exhibit a robust increase in T_4_ production that is dependent on cell seeding density (6,000 to 12,000 cells per well), microtissue size (>40 μm), and TSH concentration (0.3 and 1 mIU/mL). TSH-stimulated T_4_ production in 3D microtissues is also inhibited by prototype thyroid disrupting reference compounds, which is consistent with published potency and efficacy data. Finally, the results from this study indicate the potential utility of cryopreserved p’1 primary human thyrocytes for use in the human thyroid microtissue assay described by Deisenroth et al. (2020) and represent a suitable system to evaluate and prioritize potential TDCs for human health risk assessment.

## Data Availability

The original contributions presented in the study are included in the article/Supplementary Material, further inquiries can be directed to the corresponding author.

## Supplementary material

SUPPLEMENTARY FIGURE S1 T_4_ production correlates with microtissue diameter but not TG production. **(A)** Graph representing the linear relationship between microtissue diameter and T_4_ production. *n* ≥ 2 replicates from *n* = 5 donor lots. **(B)** Graph representing the non-linear relationship between production of TG and T_4_. R^2^ equals 0.1817. *n* ≥ 2 replicates from *n* = 7 donor lots.

## References

[B1] Andersson N. Arena M. Auteri D. Barmaz S. Grignard E. Kienzler A. (2018). Guidance for the identification of endocrine disruptors in the context of Regulations (EU) No 528/2012 and (EC) No 1107/2009. EFSA J. 16 (6), e05311. 10.2903/j.efsa.2018.5311 32625944 PMC7009395

[B2] Biondi B. (2013). The normal TSH reference range: what has changed in the last decade? J. Clin. Endocrinol. Metab. 98 (9), 3584–3587. 10.1210/jc.2013-2760 24014812

[B3] Chaker L. Bianco A. C. Jonklass J. Peeters R. P. (2017). Hypothyroidism. Lancet 390 (10101), 1550–1562. 10.1016/S0140-6736(17)30703-1 28336049 PMC6619426

[B4] Chiovato L. Magri F. Carlé A. (2019). Hypothyroidism in context: where we’ve been and where we’re going. Adv. Ther. 36 (Suppl. 2), 47–58. 10.1007/s12325-019-01080-8 31485975 PMC6822815

[B5] Citterio C. E. Rivolta C. M. Targovnik H. M. (2021). Structure and genetic variants of thyroglobulin: pathophysiological implications. Mol. Cell. Endocrinol. 528, 111227. 10.1016/j.mce.2021.111227 33689781

[B6] Deisenroth C. Soldatow V. Y. Ford J. Stewart W. Brinkman C. LeCluyse E. L. (2020). Development of an *in vitro* human thyroid microtissue model for chemical screening. Toxicol. Sci. 174 (1), 63–78. 10.1093/toxsci/kfz238 31808822 PMC8061085

[B7] Ehsani R. Alijanpour M. Salehiomran M. Kheirkhah F. Moslemi L. Aghajanpour F. (2021). Evaluation of the developmental outcome in children with congenital hypothyroidism. Casp. J. Intern. Med. 12 (3), 315–322. 10.22088/cjim.12.3.315 PMC8223043 34221282

[B8] Environmental Protection Agency (2022). Availability of new approach methodologies (NAMs) in the endocrine disruptor screening program (EDSP). Available at: https://www.regulations.gov/document/EPA-HQ-OPP-2021-0756-0002.

[B9] Faggiano A. Coulot J. Bellon N. Talbot M. Caillou B. Ricard M. (2004). Age-dependent variation of follicular size and expression of iodine transporters in human thyroid tissue. J. Nucl. Med. 45, 232–237. 14960641

[B10] Foley B. Hopperstad K. Gamble J. Lynn S. G. Thomas R. S. Deisenroth C. (2024). Technical evaluation and standardization of the human thyroid microtissue assay. Toxicol. Sci. Feb 4, 89–107. Epub ahead of print. 10.1093/toxsci/kfae014 PMC11784494 38310358

[B11] Ghanbari M. Ghasemi A. (2017). Maternal hypothyroidism: an overview of current experimental models. Life Sci. 187, 1–8. 10.1016/j.lfs.2017.08.012 28807719

[B12] Hallinger D. R. Murr A. S. Buckalew A. R. Simmons S. O. Stoker T. E. Laws S. C. (2017). Development of a screening approach to detect thyroid disrupting chemicals that inhibit the human sodium iodide symporter (NIS). Toxicol. In Vitro. 40, 66–78. 10.1016/j.tiv.2016.12.006 27979590

[B13] He J. Xu J. Zheng M. Pan K. Yang L. Wang C. (2024). Thyroid dysfunction caused by exposure to environmental endocrine disruptors and the underlying mechanism: a review. Chem. Biol. Interact. 391 (1), 110909. 10.1016/j.cbi.2024.110909 38340975

[B14] Hopperstad K. Truschel T. Wahlicht T. Stewart W. Eicher A. May T. (2021). Characterization of novel human immortalized thyroid follicular epithelial cell lines. Appl. in Vitro Toxicol. 7 (2), 39–49. 10.1089/aivt.2020.0027 PMC9157743 35663474

[B15] Hornung M. W. Korte J. J. Olker J. H. Denny J. S. Knutsen C. Hartig P. C. (2018). Screening the ToxCast phase 1 chemical library for inhibition of deiodinase type 1 activity. Toxicol. Sci. 162 (2), 570–581. 10.1093/toxsci/kfx279 29228274 PMC6639810

[B16] Jackson J. L. (1931). The shape and size of the human thyroid follicle in health and disease. Anatomical Rec. 48 (2), 219–239. 10.1002/ar.1090480202

[B17] Kalluri R. Weinberg R. A. (2009). The basics of epithelial-mesenchymal transition. J. Clin. Invest. 119 (6), 1420–1428. 10.1172/JCI39104 19487818 PMC2689101

[B18] Kendall R. T. Feghali-Bostwick C. A. (2014). Fibroblasts in fibrosis: novel roles and mediators. Front. Pharmacol. 5, 123. 10.3389/fphar.2014.00123 24904424 PMC4034148

[B19] Komur M. Ozen S. Okuyaz C. Makharoblidze K. Erdogan S. (2013). Neurodevelopment evaluation in children with congenital hypothyroidism by Bayley-III. Brain Dev. 35 (5), 392–397. 10.1016/j.braindev.2012.07.003 22858380

[B20] Männistö T. Mendola P. Grewal J. Xie Y. Chen Z. Laughon S. K. (2013). Thyroid diseases and adverse pregnancy outcomes in a contemporary US cohort. J. Clin. Endocrinol. Metab. 98 (7), 2725–2733. 10.1210/jc.2012-4233 23744409 PMC3701274

[B21] Melse-Boonstra A. Mackenzie I. (2013). Iodine deficiency, thyroid function and hearing deficit: a review. Nutr. Res. Rev. 26 (2), 110–117. 10.1017/S0954422413000061 23759468

[B22] Mueller S. Kleinau G. Szkudlinski M. W. Jaeschke H. Krause G. Paschke R. (2009). The superagonistic activity of bovine thyroid-stimulating hormone (TSH) and the human TR1401 TSH analog is determined by specific amino acids in the hinge region of the human TSH receptor. J. Biol. Chem. 284 (24), 16317–16324. 10.1074/jbc.M109.005710 19386596 PMC2713536

[B23] Noyes P. D. Friedman K. P. Browne P. Haselman J. T. Gilbert M. E. Hornung M. W. (2019). Evaluating chemicals for thyroid disruption: opportunities and challenges with *in vitro* testing and adverse outcome pathway approaches. Environ. Health Perspect. 127 (9), 95001. 10.1289/EHP5297 31487205 PMC6791490

[B24] Ochi Y. Hachiya T. Koyama Y. Fukuhori N. Ashida N. (2018). Antithyroid drugs inactivate TSH binding to the TSH receptor by their reducing action. Endocr. Metab. Immune Disord. Drug Targets 18 (5), 508–512. 10.2174/1871530318666180220101845 29468987

[B25] Paul Friedman K. Watt E. D. Hornung M. W. Hedge J. M. Judson R. S. Crofton K. M. (2016). Tiered high-throughput screening approach to identify thyroperoxidase inhibitors within the ToxCast phase I and II chemical libraries. Toxicol. Sci. 151 (1), 160–180. 10.1093/toxsci/kfw034 26884060 PMC4914804

[B26] Pirahanchi Y. Tariq M. A. Jialal I. (2023). “Physiology, thyroid,” in StatPearls (Treasure Island: FL: StatPearls Publishing). 30137850

[B27] Rousset B. Dupuy C. Miot F. Dumont J. (2000). “Chapter 2 thyroid hormone synthesis and secretion,” in Endotext. Editor Feingold K. R. Anawalt B. Blackman M. R. Boyce A. Chrousos G. Corpas E. South Dartmouth (MA: MDText.com, Inc). 25905405

[B28] van Staveren W. C. Solís D. W. Delys L. Duprez L. Andry G. Franc B. (2007). Human thyroid tumor cell lines derived from different tumor types present a common dedifferentiated phenotype. Cancer Res. 67 (17), 8113–8120. 10.1158/0008-5472.CAN-06-4026 17804723

