# Supplementary figures and images for: Characterization of a human thyroid microtissue model for testing thyroid disrupting chemicals
Source: Front Toxicol. 2024 Jul 24;6:1408808. doi: 10.3389/ftox.2024.1408808 (PMC11303298; doi:10.3389/ftox.2024.1408808)

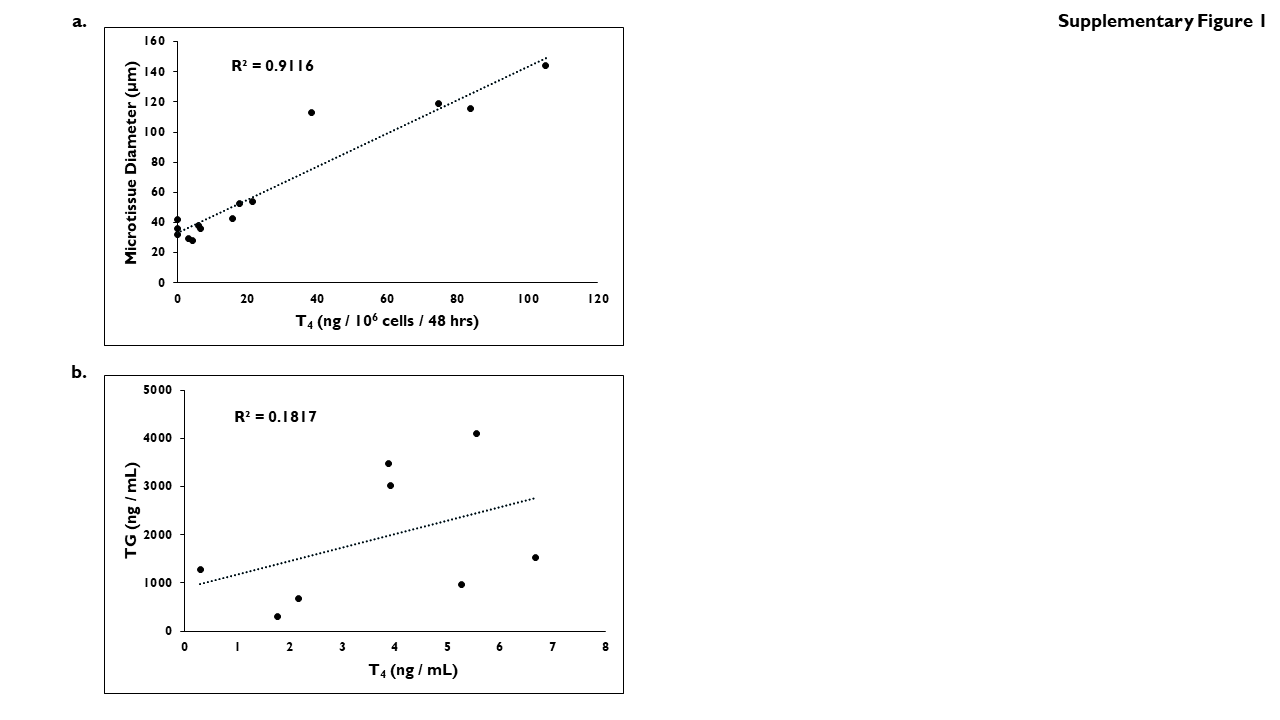

Supplement: Supplementary file 1 [file Image1.tif]
